# ‘Massive potential’ or ‘safety risk’? Health worker views on telehealth in the care of older people and implications for successful normalization

**DOI:** 10.1186/s12911-016-0373-5

**Published:** 2016-10-12

**Authors:** Wendy Shulver, Maggie Killington, Maria Crotty

**Affiliations:** 1 Rehabilitation, Aged and Extended Care, Flinders University, Daws Road, Daw Park, Adelaide, South Australia Australia; 2 Rehabilitation and Aged Care, Repatriation General Hospital, Adelaide, South Australia Australia

**Keywords:** Telehealth, Implementation, Normalization, Normalization process theory, Qualitative, Rehabilitation, Aged care, Palliative care, Allied health

## Abstract

**Background:**

Telehealth technologies, which enable delivery of healthcare services at distance, offer promise for responding to the challenges created by an ageing population. However, successful implementation of telehealth into mainstream healthcare systems has been slow and fraught with failure. Understanding of frontline providers’ experiences and attitudes regarding telehealth is a crucial aspect of successful implementation. This study aims to examine healthcare worker views on telehealth, and their implications for implementation to mainstream healthcare services for older people. The study includes a focus on two further dimensions of urban versus rural services and level of clinician experience with telehealth.

**Methods:**

Seven semi-structured focus groups were conducted with a total of 44 healthcare workers providing services to older people in the areas of rehabilitation and allied health, residential aged care and palliative care. Focus groups included both telehealth experienced and inexperienced groups. Of the experienced groups, two provided services to both urban and rural patients, and two to rural patients. Inexperienced groups included one rural and two urban. Thematic analysis was undertaken to identify predominant themes. Between-group differences and agreement in viewpoints for each of these themes are discussed and mapped to the theoretical constructs of Normalization Process Theory.

**Results:**

The views of participants varied with the extent of telehealth experience and perception of accessibility of healthcare services. Four themes describing clinician attitudes and perceptions that could impact on successful implementation of telehealth services are outlined: 1) Workability of telehealth: exponential growth in access or decay in the quality of healthcare? 2) What is an acceptable level of risk to patient safety with telehealth? 3) Shifting responsibilities and recalibrating the team; and 4) Change of architecture required to enable integration of telehealth service delivery.

**Conclusions:**

The use of telehealth technologies to provide healthcare services to older people may be more readily normalized in areas where existing services are limited. Though exposure to telehealth may be a factor, changes to the perceived feasibility of telehealth in relation to conventional services, as well as supportive infrastructure and training and skill recalibration may be more critical to successful normalization of telehealth services for older people.

## Background

The use of telehealth technologies to deliver healthcare services at distance has been promoted as a promising and cost-effective way to address the challenges created by the healthcare needs of an ageing population [1, 2]. Rehabilitation and palliative care are key services for older people as most recipients are aged above 65 [3]. Telehealth technologies offer promise for increasing dosage of exercises for older people undergoing rehabilitation following such events as hip fracture and stroke, which is associated with better outcomes [4–8]. Other potential advantages of service delivery to older people via telehealth include reduction of potentially distressing travel for people such as palliative care patients and people with dementia, and better outreach of specialist services into residential aged care facilities. Defined as the ‘remote exchange of data between a patient and healthcare professionals as part of the patient’s diagnosis and healthcare management’ [1], telehealth employs telecommunication technologies to enable transfer of information in the form of voice, data and images between patients and healthcare providers.

While telehealth interventions have been shown to improve clinical indicators, successful implementation and adoption of telehealth has been slow and fraught with failure. Many telehealth services remain at the status of ‘innovation’, not extending beyond research pilots or niche markets to become part of routine healthcare delivery [1, 9, 10].

Prior work examining implementation of telehealth has identified factors such as infrastructure, technological issues, change management, jurisdictional and organizational boundaries and funding that may impact on successful integration of telehealth services [11–13]. The differing interests and perspectives of various stakeholders including patients, health professionals, managers, policy makers and information technologists are also important [12–15]. While understanding direct providers’ experiences and attitudes regarding telehealth services is a crucial aspect of successful implementation, indepth details on how health professionals view telehealth and their roles in the introduction and provision of telehealth services remains under explored [14, 16, 17].

In Australia, a universal healthcare system is largely funded by the Federal government, with service provision, including public hospitals and regional health networks, administered and run by state governments. The Australian federal government recently funded a Telehealth Pilots Programme, aiming to develop, deliver and evaluate telehealth services to patients’ homes, with a focus on aged, palliative and cancer care services. The Flinders University of South Australia received funding to undertake a trial of aged and palliative care services delivered via telehealth, in partnership with a public hospital, the local rural health network and an aged care provider in South Australia. The hospital serves a local catchment area of Adelaide, South Australia, including provision of rehabilitation, aged, allied health and palliative care services. The qualitative work presented in this paper was undertaken as a component of this trial.

The aim of the present study is to examine healthcare worker views on telehealth, and their implications for integration of telehealth into mainstream healthcare services in the care of older people. Within this broad aim, the study examines two further dimensions that the literature suggests can impact and drive implementation of telehealth services, namely the urban/rural divide and level of clinician experience with telehealth. There is an expectation that telehealth technologies will deliver greater access to healthcare for rural and remote populations by enabling delivery of healthcare to people in their home locations [10]. We also wanted to explore in this study the acceptability to clinicians of using telehealth in urban areas. Clinician acceptance has been highlighted as a key aspect of successful implementation of telehealth interventions, and experience with telehealth has been shown to impact on such acceptance [17–20]. Thus the study aims to answer the following questions: 1) What are the views of healthcare workers providing services to older people on telehealth?; 2) Are there differences in these views between healthcare workers providing services in rural versus urban areas?; 3) What impact does level of experience with telehealth have on healthcare workers’ views on telehealth?; and 4) What are the implications of these views and attitudes for the successful implementation of telehealth in the provision of services to older people? The study reports on the views of healthcare workers providing services in both rural and urban areas, and who have a range of telehealth experience. Normalization Process Theory, which was developed to enable examination of implementation and integration processes of complex healthcare interventions, was used as the theoretical framework.

### Normalization process theory

Normalization Process Theory (NPT) [21, 22] is a middle range sociological theory that can be used to understand the factors that facilitate or inhibit the implementation and embedding, or ‘normalization’, of complex healthcare interventions into routine practice. There are numerous implementation frameworks that have been developed for or applied to telehealth services, including NPT [12, 23–25]. NPT enables multifaceted examination of the complex and inter-related factors that can impact implementation of telehealth interventions, rather than focusing on particular aspects [18]. It is a useful framework for analysis of frontline healthcare worker experiences as it focuses on the work that people do, both individually and collectively, to integrate a complex intervention into practice, and contends that successful integration requires continuous investment in this work. The theory proposes that implementation occurs via four generative mechanisms which are affected by factors that facilitate or inhibit normalization of an intervention. These mechanisms are:

*Coherence –* the way in which actors make sense of and understand an intervention. Coherence requires that actors reach a shared understanding of how the practice is defined and differentiated from other practices.

*Cognitive Participation –* actors’ engagement with a new practice. Successful integration depends on the enrolment of actors to create a community of practice, and their legitimation of the practice.

*Collective Action –* the practical work of integration. This work is governed by two modes of interactions between people: *interactional workability* (congruence of interactions between health professionals and patients) and *relational integration* (working knowledge required by users and confidence in the broader network in which their work is situated). Organizing conditions that impact on these interactions are *skill-set workability* (the division of labour and allocation of specific tasks related to an intervention and the calibration of these tasks to required skill-sets) and *contextual integration* (the fit of the intervention within existing organizational structures, systems and practices).

*Reflexive Monitoring –* participants’ ongoing evaluation, both formally and informally, of a practice and the implementation process.

## Methods

**Fig. 1:**
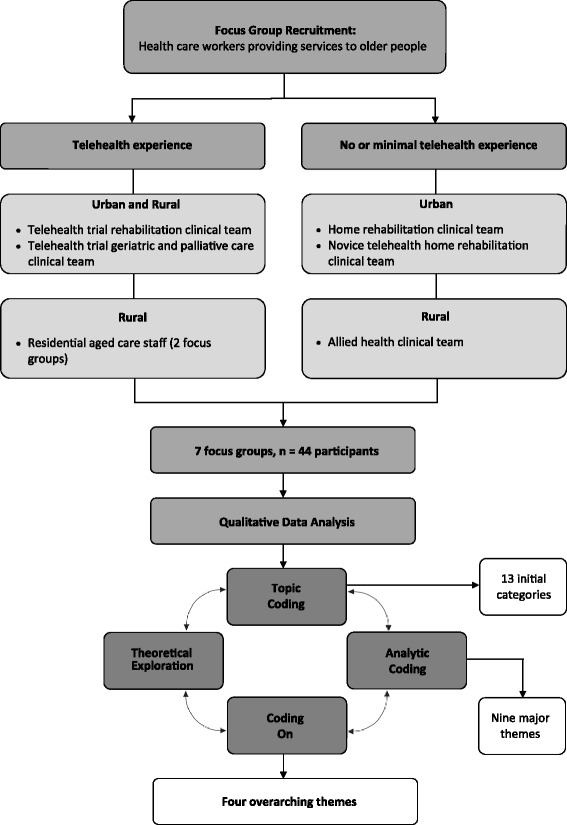
Methodological schema

Seven focus groups were conducted with clinicians and care workers providing services to older people in the areas of rehabilitation and allied health, aged care and palliative care to rural and urban areas. For the purposes of this study, ‘rural’ was defined as the areas serviced by the local rural health network, and ‘urban’ was defined as areas within the greater Adelaide (capital city of South Australia) region. All focus groups were ‘natural groups’, in that they comprised of clinicians and care worker teams that already knew and worked with the other participants in the group [26]. Figure 1 provides an overview of the study methodology, including focus groups recruited, number of focus groups and participants, and the data analysis processes. Table 1 provides further information about the specific services provided by participants, their level of experience with using telehealth for providing patient care and the type of areas they served (i.e., urban or rural). All participants worked for either State government health networks or aged care providers. As shown in Fig. 1 and Table 1, the study included participants serving rural areas both with and without telehealth experience, and similarly included participants serving urban areas both with and without telehealth experience. The experienced group provided services to both urban and rural areas, and incorporated clinicians and residential aged care facility staff involved in the larger telehealth trial testing telehealth as a way of delivering rehabilitation, geriatrics and palliative care in Southern Adelaide, South Australia. Both the trial and qualitative study were approved by the Southern Adelaide Clinical Human Research Ethics Committee, reference number HREC/13/SAC/121 (203.13).

**Table 1:** Focus group participants, level of telehealth experience and service provision area (urban or rural)

Participants	Level of telehealth experience in patient care	Health services provided to older people	Focus group details
Participants providing services to urban areas			
Urban ambulatory rehabilitation in the home clinical team: ‘urban clinicians’	None	Face-to-face physiotherapy (PT), Occupational Therapy (OT), Social Work (SW), Exercise Physiology (EP), Speech Pathology (SP), Rehabilitation Nursing (RN) in patients’ homes in southern urban Adelaide.	Focus group 1 13 participants Face-to-face at urban hospital
Urban ambulatory rehabilitation in the home clinical team post implementation of telehealth into the service: ‘novice telehealth urban clinicians’	Minimal	Face-to-face PT, OT, SW, EP, SP, RN in patients’ homes in southern urban Adelaide. Encouraged to provide some services at distance via telehealth following roll-out of the service post-trial. Clinicians exercised their own discretion regarding how much of their caseload they used telehealth to provide their service, with no set criteria for assigning a patient to telehealth delivered services.	Focus group 2 9 participants Face-to-face at urban hospital
Participants providing services to rural areas			
Residential aged care team: ‘residential aged care staff’	6 months	Supported residents who participated in telehealth geriatric review and rehabilitation at two rural residential aged care facilities. Review, assessments and follow-ups conducted via specialist videoconferencing equipment installed at the aged care facility.	Focus group 3 6 participants Face-to-face at residential aged care facility Focus group 4 2 participants Videoconference
Rural rehabilitation allied health clinical team: ‘rural allied health clinicians’	None	Rehabilitation and allied health services to rural areas in South Australia.	Focus group 5 7 participants Teleconference
Participants providing services to both urban and rural areas			
Telehealth trial clinical team: ‘telehealth clinicians’	6 months	Service provision via telehealth as part of a telehealth in the home trial: *Rehabilitation*: Combination of face-to-face and distant PT, OT, SP assessment, intervention and review, distant activity monitoring. To community patients in urban areas of Adelaide, and rural patients living in residential aged care. Monitoring done via provision of ‘off-the-shelf’ (i.e., iPad) technology to patients in their homes, and specialist videoconferencing technology installed in residential aged care facilities. Activity level data was electronically transmitted and assessments and follow up conducted via videoconference. *Geriatric review* for patients living in rural residential aged care facilities: Specialist videoconferencing technology installed at the facility through which a geriatrician located at the hospital conducted distance reviews with the support of a ‘trial nurse’ and facility staff ‘on the ground’. *Palliative care* to community patients: Combination of face-to-face and distant intervention, including daily self-reporting of patients’ symptoms via iPad provided to the patient in their home.	Focus group 6 5 participants Face-to-face at urban hospital Focus group 7 2 participants Face-to-face at urban hospital

The larger telehealth trial also included a quantitative Discrete Choice Experiment and attitudinal survey designed to assess older peoples’ preferences and attitudes regarding telehealth. The focus group data presented here was collected as part of this nested study, to be used initially for development of the questionnaire [3]. The quantitative survey project guided both the selection of groups for inclusion in the focus groups (rehabilitation, aged and palliative care clinicians/careworkers who were or may in the future provide telehealth services) and the focus group topic guide. The topics and sampling methods were therefore not theoretically derived. However, the sample included all groups within South Australia that could have reasonably been targeted for such a topic, including all clinician groups participating in the larger trial. Moreover, the content of the focus groups provided excellent data on clinician attitudes, and valuable insights enabling useful theoretical exploration of implementation factors with regard to healthcare services for older people. On reading the NPT literature in conjunction with our preliminary analysis of the focus group data, there was indeed resonation between data and theory, warranting deeper analysis using NPT as a useful conceptual framework.

Four of the seven focus groups (urban clinicians, novice telehealth urban clinicians and two telehealth clinician groups) were conducted at the urban hospital in which they worked, and one residential aged care group was conducted at the participating rural aged care facility. All focus groups that were able to be conducted face-to-face were done so, however, due to logistical and distance difficulties, it was necessary to conduct two focus groups with rural health service providers at distance (one via videoconference with a second rural residential aged care facility and one via teleconference with rural allied health clinicians). The potential disadvantages of these synchronous technologically mediated focus groups, such as lack of non-verbal information in the case of teleconference groups, were far outweighed by the key advantage of enabling these health workers to participate thereby strengthening the sample and maximising the capture of a broad range of experiences and views [27]. Given that in this study the participants were motivated and engaged professionals with sufficient technological expertise, and that the questions and topics covered in these groups were the same as for the face-to-face groups, we posit that the impact of differences in focus group methodology on results is unlikely to be significant.

The number of participants in focus groups ranged from two to 13, with a total of 44 participants. Each focus group took between half to one hour. Focus groups with urban clinicians and novice urban telehealth clinicians included the clinical team and their manager. These focus groups were conducted at the time the team held their regular team meeting. All participants had the study described to them and were informed that participation was voluntary. All present at the meeting consented to participate. All ‘telehealth’ clinicians involved in the trial were invited to participate in a focus group and consented to do so. No trial clinician declined participation. Rural allied health participants included senior clinicians from each of the allied health disciplines. They were contacted through the chief allied health officer for the local rural health network. All who were contacted agreed to participate. The nurse managers of the residential aged care facilities who participated in the telehealth trial were contacted regarding the qualitative study and asked to participate in a focus group with nursing and caring staff who had been involved in the trial. All staff who were available at the time of the focus group agreed to participate. The focus groups were moderated by the researcher MK with the assistance of WS. MK was involved in the design, ethics approval and start-up of the larger telehealth trial. She was therefore known to the participants working on the trial, but did not have a direct working relationship with them and was not directly involved in patient care.

Experienced healthcare workers discussed ‘telehealth’ with reference to their practice during the trial, as summarised in Table 1. Novice telehealth clinicians were beginning to utilise telehealth technology in the same way as used in the trial for rehabilitation patients following roll-out of the service post-trial. Telehealth was defined at the beginning of focus groups with inexperienced clinicians as ‘the use of telecommunication technologies to provide healthcare services. These services enable passing of information in the form of voice, data and images between patients and health professionals’. Examples were provided of ways in which telehealth technology can be used. All participants without direct experience indicated an understanding of telehealth and what it can be used for in terms of service delivery and patient care.

The focus groups were semi-structured. Topics covered included asking participants to describe their telehealth experiences, the clinical areas (if any) in which they have used the technology, their views on the positive and challenging aspects of providing healthcare via telehealth, challenges and requirements for the implementation of telehealth services, benefits, compromises, quality of care with telehealth and any other input they had regarding telehealth. Probing was used to ensure topics were sufficiently explored. Each focus group was challenged until no more new information or perceptions were forthcoming about the topic.

Interviews were recorded and transcribed verbatim. NVivo10 qualitative data analysis software was used as a data management tool to aid the analysis. Thematic analysis was undertaken to identify predominant themes in the data. Analysis was based on the coding procedures described by Richards [28]. The researcher MK undertook the first analysis stage of ‘topic coding’, involving line-by-line coding of each transcript to develop a provisional coding framework consisting of 13 initial categories. The initial analysis and resulting coding framework was descriptive rather than conceptual at this stage, and all transcripts were analysed together, without examination of the differentiation in responses between different clinician groups. In the second coding phase of ‘analytic coding’ further, more indepth analysis was undertaken to develop conceptual ideas. MK and WS examined and discussed the provisional codes to draw out and agree on nine predominant themes. In the final ‘coding on’ stage the themes were re-examined, with particular attention to any patterns, similarities and differences in responses between the different groups. It was found that there was a high level of agreement within groups and some clear differences in views between groups. Though our study included the viewpoints of a range of clinician groups, we did not make specific assumptions about between-group differences in our preliminary analysis. However it became apparent through the analysis that between-group comparison was a fruitful method of making sense of the factors that impact on successful implementation of telehealth services. These differences and their theoretical resonance were reflected on and discussed, resulting in the nine subthemes collapsed into four overarching themes. These were mapped to NPT to further develop ideas regarding the implementation potential of telehealth between the different groups, and the likely promoting or inhibiting factors. For example, the overarching theme regarding acceptable risk derives from subthemes outlining the differences between experienced clinicians’ strategies to overcome risk and inexperienced urban clinicians’ concerns about compromising safety with telehealth. MK and WS each worked on the development, writing up and mapping of individual themes to theoretical concepts, with regular meetings to discuss and refine their development. Though we have described the analysis in distinct stages for clarity, consistent with qualitative research in general, analysis in practice was not linear but rather an iterative process, with continual cycling between coding stages and theoretical exploration.

## Results

The four overarching themes are outlined below, within each of which we highlight the contrasting viewpoints of different groups. Table 2 summarises each group’s position on each of the four themes. Illustrative quotes are tagged with the focus group name, number and level of telehealth experience. Results are linked to the theoretical concepts of NPT in the discussion, and the implications for implementation of telehealth services for older people outlined.

**Table 2:** Health worker positions on each of the 4 identified themes

Theme	Urban clinicians	Novice telehealth urban clinicians	Residential aged care staff	Rural allied health clinicians	Telehealth clinicians
Workability of telehealth: exponential growth in access or decay in the quality of healthcare?	Reservations about the safety and suitability of telehealth and the limitations it places on what they can do at distance. Better than nothing for people in rural areas who cannot easily access face-to-face services	Significant portion of caseload could be serviced via telehealth, but similar reservations to urban clinicians, particularly for complex cases	Positive about the effectiveness of telehealth and saw it as just as good as face-to-face	‘Massive potential’ to expand services and provide better access to healthcare to rural locations	Positive about the potential of telehealth and keen to explore possibilities the technology could offer to enhance and expand their services
What is an acceptable level of risk to patient safety with telehealth?	Concerned about the levels of perceived risk with telehealth, associated with not being with the patient to assist in the event of an adverse advent (for example a fall during exercise)	Revert from telehealth back to face-to-face if complications arise, but acknowledged that ongoing experience can promote new ways of managing challenges of telehealth	Focussed on perceived improvements in outcomes for aged care residents who had received services via telehealth, rather than risk to their safety	Focussed on the potential improvements to patient outcomes through better access to services, rather than risk to patient safety. Telehealth is ‘safe’ and ‘equivalent, if not better’ than conventional face-to-face therapy	Accepting and pragmatic about risk, which they thought of as something to be planned for and managed as an integral part of the provision of services via telehealth
Shifting responsibilities and recalibrating the team	‘Risk’ problems with telehealth could be alleviated by having a support person ‘on the ground’ with the patient.	‘Risk’ problems with telehealth could be alleviated by having a support person ‘on the ground’ with the patient	Took on the role of ‘on the ground’ support during videoconferences with residents of the aged care facilities, and through this increased their skill levels	Keen to forge links with urban speciality services to support rural clients	Adequate training of ‘on the ground’ supporters is important
Change of architecture required to enable integration of telehealth service delivery				Existing ‘traditional’ organizational and systemic structures need significant overhaul before being able to fully support outreach telehealth services	Concerns about the limitations of existing technological infrastructure and support. Keeping up with rapidly changing technology and the required technological training and support will be challenging

### Theme 1: Workability of telehealth: exponential growth in access or decay in the quality of healthcare?

Both rural allied health and telehealth clinicians were positive about the potential of telehealth and very keen to explore any possibilities the technology could offer which might enhance and expand access to the services they were able to deliver. Rural health clinicians strongly voiced their enthusiasm that it would support the clinical needs of their patients despite having little or no experience of using the technology:

*I think there’s a massive potential to expand the sorts of services we can provide to people in their local communities by stronger partnership with metropolitan* [urban]-*based specialty services linking with our general clinicians to support the client on the ground* [rural allied health clinician, FG 5, no experience].

The rural allied health team indicated that telehealth technology provided ‘a whole range of other capabilities’, and considered it ‘safe and it’s appropriate and it’s an equivalent, if not better, sort of service that you can provide’. They were committed to the notion that telehealth could balance the unequal access to services across geographical locations, and were keen to pursue innovative ways of using telehealth technologies to allow them to provide complex distant therapy.

In contrast to rural and experienced telehealth clinicians who were keen to utilise technology as part of their role and to deal with distance and isolation, urban clinicians with no exposure to telehealth reported more reservations about the safety and suitability of providing rehabilitation through telehealth. They generally felt that telehealth should be reserved for ‘people who are more autonomous and more capable and … straightforward’, rather than ‘real’ rehabilitation patients with complex issues. They felt that people who required rehabilitation often require a ‘hands on’ approach:

*I like to be a lot more hands on with those people, particularly when there are sensory deficits and if there are compounding issues with communication and things like that, I know I would have my reservations about using telerehab* [rehabilitation via telehealth] *in those circumstances* [urban clinician, FG 1, no experience].

In addition, urban clinicians were concerned that reduced access to the patients’ home would adversely limit the information that they could collect and potentially increase risks to the patient:

*Once you’re actually there and you can see the entire house that gives you a much better picture. There are so many visual cues that you get from being in the room and being in the home. Smell, cleanliness, dishes, people’s self-care, their family, social interactions* [urban clinician, FG 1, no experience].

Urban clinicians perceived such risks in relation to their own urban patients. However, with regard to rural areas with limited access to services, their views became more aligned with those of rural health clinicians. For rural patients, they considered that compared to no intervention, telehealth delivery became more acceptable:

*I think in the rural sector, it’d be great, for people who can’t access services … It’s probably a little different in the metropolitan* [urban] *area* [urban clinician, FG 1, no experience].

Novice urban clinicians, who had very recently commenced utilising telehealth technology in the provision of their services, were somewhat more accepting of telehealth than their colleagues who had not been exposed. They generally agreed that a significant proportion of their caseload could be serviced via telehealth, however they retained reservations about the telehealth approach, particularly for more severely impaired patients:

[Telehealth] *is a great way of treating functionally declined patients who need to improve their strength and endurance … fantastic for them. It is probably not so good for some of the stroke hands on type manual therapy type of … patients* [novice telehealth urban clinician, FG 2, minimal experience].

Unlike urban clinicians, staff working in the aged care facilities which received a telehealth service were positive about the effectiveness of rehabilitation via telehealth and noted many changes in residents, such as increased involvement in activities and taking responsibility for their mobility. When asked whether they would have preferred more face-to-face input, rather than videoconferencing as provided, residential aged care staff unanimously answered in the negative:

*I mean, of course, it is nice to have a face-to-face, but I think the teleconference was just as good. It just felt like* [therapist] *was with us* [residential aged care staff, FG 3, 6 months experience].

### Theme 2: What is an acceptable level of risk to patient safety with telehealth?

Discussion of ‘risk’ in the focus groups centred on the risks to patients associated with providing services at distance. Participants recognised that things could go wrong, for example a patient falling during exercises or choking during a swallowing assessment. Urban clinicians were concerned at the levels of perceived risk with telehealth, associated with not being with the patient to assist in the event of an adverse event. In contrast, the experienced telehealth trial clinicians were more accepting of and pragmatic about risk. They tended to think of risk and patient safety as something to be planned for and managed as an integral part of the provision of services via telehealth. Rather than something that would prevent or severely limit what could be done via telehealth, different and alternative ways of thinking about and managing risk due to the absence of physical proximity to the patient were required. As one clinician put it, ‘it’s challenging what we previously have thought “well you have to be there to do”’:

*You have to calculate the risk. Really you couldn’t just decide on the spur of the moment, exercises that you’re going to do. You have to plan a bit more because of that risk-taking and maybe have some* [safety] *parameters in place for patients* [telehealth clinician, FG 6, 6 months experience].

Novice urban clinicians showed a similar approach to risk as their inexperienced counterparts. Though open to the idea of using telehealth for patient care, their response to cases in which a complication or challenge arose was reversion back to conventional face-to-face therapy:

*If I chose to do the teleconferencing* [telehealth]*, I might have to leave myself enough time and then if something* [a complication] *came up and … I felt as though I need to actually follow that up today or tomorrow if I had the time to go out and see them in person* [novice telehealth urban clinician, FG 2, minimal experience].

However, there was also acknowledgement in this group that ongoing experience can promote development of new ways of practice and ways of managing the inherent challenges of providing services via telehealth:

*So I think ultimately, with experience we get to know more about maybe ways around that but there is quite a lot* [of therapy] *that is hands on as well* [novice telehealth urban clinician, FG 2, minimal experience].

### Theme 3: Shifting responsibilities and recalibrating the team

There was a sense among all groups that many ‘risk’ problems associated with telehealth could be alleviated by having someone with the patient ‘on the ground’ to support distance healthcare via videoconference (i.e., a carer, residential aged care staff, family member, local health professional):

*I wouldn’t do a swallowing assessment without someone in the home because I need someone there to either be looking in the mouth or shining a light into their mouths … and to be there if there is a choking incident* [telehealth clinician, FG 6, 6 months experience].

Residential aged care staff clearly took on supportive roles during videoconferences with residents. This was seen in a positive light, with these new responsibilities providing opportunities to learn and upskill, and greater job satisfaction:

*It’s really helped us to know a lot of things with massaging them and just new ideas with exercises and using the weights* [residential aged care staff, FG 3, 6 months experience].

A service model that incorporates collaboration with local health professionals was considered an important aspect of successful provision of service via telehealth among all focus groups:

*I’d be looking at a local health professional, tapping into their view, getting them to knock on the door … Because there needs to be a collaborative thing if we’re using telehealth to support them, we need to work with the local people* [telehealth clinician, FG 7, 6 months experience].

However, telehealth clinicians recognised that such shifting of tasks and responsibilities requires training and recalibration of skill-sets between telehealth providers and ‘on the ground’ supporters to enable them to undertake this role:

*Neurological and physical assessments would be quite difficult because you’d have to rely on the person or a carer at the other end to be able to move that person in a way that would give you enough information and you get a lot of information from the way something feels …You could possibly do it, but you’d have to be confident that there’d be prior training of the carer to help with that or to give that sort of feedback* [telehealth clinician, FG 6, 6 months experience].

*I think it would probably need to be someone trained that I would feel confident could manage a choking incident, for example, so I guess that would need to be discussed with management, is the carer sufficient or would it need to be someone else - just for risk management* [telehealth clinician, FG 6, 6 months experience].

### Theme 4: Change of architecture required to enable integration of telehealth service delivery

Rural health clinicians in particular thought that existing organizational and systemic structures, which were ‘very traditional in their approach’ would need significant overhaul before being able to fully support outreach telehealth services to rural areas. These issues ranged from jurisdictional barriers, funding structures and changes to referral pathways and follow-up procedures:

*But if we’re talking about comprehensive healthcare and our patient journey and that whole follow through of supporting people in the context in which they live and work and all that sort of thing, then I think there are limitations the more remote from the area of the service. That’s not to say it can’t be done, but because our health services are largely jurisdictional … There would be some bureaucratic processes that would need to be worked through around sharing of information and patient flows and that knowledge of expectation of service response if you recommended or referred - all of that sort of stuff* [rural allied health clinician, FG 5, no experience].

Concerns were also raised by telehealth clinicians about the quality and range of the telehealth infrastructure currently available to them, and the limitations this places on what can be done via telehealth. Given access to more technology and support, they felt that they could further expand the telehealth services they can provide:

*I think we’re limited with the range of technologies that are available to us … There are a lot of things that we can’t do at the moment, but it’s not because it’s not possible, it’s because we don’t have the technological support. For example, a second camera or a split-screen availability to make sure that we’re showing the person the right things* [telehealth clinician, FG 6, 6 months experience].

Ongoing technical support was also raised as a factor that could impact successful implementation of telehealth services. Telehealth clinicians felt that successful embedding of a telehealth service requires organizational provision of comprehensive technical support, beyond simple helpdesk support:

*I think there has to be some sort of service that goes beyond pure IT support around logging on/logging off type stuff. I think there has to be maybe a new stream of either therapist or parallel to that are technicians that actually have a clinical focus that can actually drive this and I don’t think we have that at the moment* [telehealth clinician, FG 7, 6 months experience].

It was also recognised that rapid and ongoing advances in technology will have an ongoing impact on telehealth service structures and pose challenges for the maintenance of the working knowledge required to effectively use telehealth:

*We are dealing with areas that are ever-changing and requiring support and I think that is going to be one of our huge limitations to providing this type of service in the future, because whilst we will all up-skill and we will all get better at using it because we just use it in our everyday lives, I think it will then change and we’ll get a new machine or a new game or a new program which we will then have to* [learn] *… So it’s rapidly changing and I think that is the challenge for us all to know what’s out there and what’s going to help us* [telehealth clinician, FG 6, 6 months experience].

## Discussion

This study contributes to the insufficiently understood area of how health professionals view their roles in telehealth service provision, and furthers our understanding of the implementation potential of telehealth in the context of care services for older people in Australia. A multifaceted examination of these issues, through the use of NPT as a conceptual framework, has to our knowledge not previously been undertaken. A 2014 systematic review of studies using NPT to examine implementation processes found a number of studies examining telehealth/telecare/e-health initiatives, but none specifically concerned with the service areas of rehabilitation, aged or palliative care [29]. Subsequent relevant NPT studies have looked at secondary hip fracture prevention services, and decision making for people with dementia in Australian aged care facilities [30, 31], but again, none have looked at the specific contexts examined here. Our results show a contrast in the attitudes of rural and telehealth experienced healthcare workers about telehealth, compared to urban inexperienced clinicians. Table 3 summarises each clinician group’s views on telehealth by the NPT generative mechanisms. In NPT terms, there was *coherence* in the way rural and telehealth clinicians defined and understood telehealth. They clearly differentiated telehealth as a distinct model of service that required new ways of working. These clinicians were focussed on the potential for telehealth to achieve better outcomes for patients, and were willing to re-think and adjust their practice to provide distance healthcare. In contrast, inexperienced and novice urban healthcare workers had not yet developed *coherence* with regard to telehealth. They did not conceptualise telehealth as a distinct model of service, but rather as an adjunct to conventional services, as evidenced by their perception of telehealth services differently for urban and rural patients. Nor were they embracing and thinking about novel solutions to risks and challenges in providing their services via telehealth, but rather felt that face-to-face service delivery is the best, and in complex cases, the only appropriate method.

**Table 3:** Summary of views on telehealth by participant group and NPT generative mechanisms

Clinician group	Coherence	Cognitive participation	Collective action
Urban clinicians	Low. Perception of telehealth did not cohere with that of other groups of clinicians. Perceived the service differently for rural than for urban patients.	Low. Do not see telehealth as a legitimate model of service for ‘real’ rehab patients (particularly urban patients).	Interactional workability – low. Only suitable for high-functioning or remote patients (who have no option for face-to-face consults). Patient-therapist interactions would suffer due to narrowing of the scope of what can be seen and done via videoconference. Relational integration – low. Focussed on current patients rather than wider service delivery limitations
Rural allied health clinicians	High. Clearly defined, differentiated and understood telehealth and its potential	High. Willingness to engage with telehealth as a model of service; viewed telehealth as a legitimate way to expand their services; looking at ways to create a community of practice utilising local health professionals.	Interactional Workability – High. Promising way to provide access to services for rural patients who lack local services. Relational integration – High. Clear understanding of the wider health network and could see opportunities for using telehealth to link urban and rural services Contextual integration: Low Considerable organization, systemic and technological infrastructure barriers
Novice telehealth urban clinicians	Developing. Telehealth still conceptualised as an adjunct to traditional model of service.	Developing. Burgeoning acceptance of telehealth but retained concerns about the efficacy of telehealth for more severely impaired patients.	Interactional workability – low to moderate. Agreed that a significant proportion of work could be done via telehealth but still felt that it was not suitable for significantly impaired patients. Relational integration – low. Conventional model of service seen as ‘core business’, with telehealth incorporated at their discretion, rather than embracing novel ways of working using telehealth.
Telehealth clinicians	High. Clearly differentiated telehealth as a distinct model of service that required new ways of working.	High. Engaged with the service and were thinking about ways to expand its scope and make it work	Interactional workability – moderate. Careful planning and improvements in technology required to maximise what can be done via telehealth Skill-set workability – questionable. Concerned that on the ground supporters of videoconferences adequately trained. Relational integration – moderate to high Concerns about keeping up with rapidly changing technology to maintain working knowledge; forged supportive networks with residential aged care facilities Contextual integration – Moderate Technological infrastructure and tech support required Reflexive monitoring – High. Re-conceptualised telehealth as a distinct model, rather than an adjunct to traditional models. Thinking about ways in which they could improve, expand and respond to challenges in providing their services via telehealth
Residential aged care staff	High. Positive about the impacts of telehealth as a new service not previously available to their residents	High. Embraced the service and collectively enrolled.	Interactional workability – High. Positive outcomes for residents. Teleconference as good as face-to-face. Relational integration – High. Established links with urban rehabilitation and geriatric services. Skill-set workability – High. Displayed the skills to support interventions; increased their skill-set through doing so.

Rural and telehealth experienced healthcare workers also demonstrated *cognitive participation* in clearly viewing telehealth as a legitimate way to expand their services and showing a willingness to engage with it, and establish a community of practice by working together and forging links with other services and clinicians. *Cognitive participation* is less developed among inexperienced urban clinicians with respect to urban patients as many do not see it as a legitimate service model for ‘real’ patients (i.e., those with complex issues). Similarly, novice urban clinicians, whilst more open to the prospect of increasing utilisation of telehealth than those with no experience, still perceived these same limitations.

There were also differences between rural and telehealth clinicians and both novice and inexperienced urban clinicians in terms of *collective action*. In particular, urban clinicians were concerned that telehealth compromises *interactional workability*. They did not believe that telehealth is an appropriate substitute for face-to-face interactions with patients who they would otherwise see in person and felt that the patient-therapist interaction and its outcomes would suffer due to a perceived narrowing of the scope of what they can see and do over videoconference. By contrast, experienced telehealth clinicians and residential aged care staff demonstrated successful interactions between clinicians and patients via telehealth, though telehealth clinicians felt that they could further improve interactions with access to better technology. Rural allied health clinicians and residential aged care staff perceived an *interactional advantage* of telehealth in its potential to accomplish improved disposal of work and access to healthcare for the rural populations they served*.* Aged care facility staff saw no compromise to *interactional workability*, believing that videoconference interactions were equal to face-to-face.

Telehealth clinicians and aged care facility staff were able to establish *relational integration* of the telehealth service by gaining the knowledge required to support the service and forge links between urban based rehabilitation and geriatric services and rural aged care facilities. Rural allied health clinicians had a clear understanding of the wider health network in which they practiced and the limitations of existing services, and could see opportunities to maximise *relational integration* of services by using telehealth to link urban and rural healthcare services.

The willingness to engage and utilise other health professionals shown by all groups, in particular experienced, rural and residential aged care participants, holds promise for *cognitive participation*, the enrolment of others towards the development of a telehealth community of practice, and *relational integration* towards an integrated team of clinicians and carers working at distance. However, this study highlights the importance of monitoring the *skill-set workability* of such a network. Residential aged care clinicians did not experience difficulties adapting their skills to enable them to support rehabilitation and geriatric assessment videoconferences on the ground. They felt, in fact, that their skills were enhanced through their participation in the trial. Nevertheless, the skill-set workability of ‘on the ground’ supporters was raised by telehealth clinicians, who recognised that telehealth may require enlisting new sources of support and recalibration of skills in order to achieve effective telehealth services.

As argued by Mort et al., through telehealth, ‘responsibilities in care networks are shifted and delegated in new ways’ [32]. Reliance of others ‘on the ground’ to support telehealth service delivery has the effect of shifting the burden of care work and re-allocating tasks and responsibilities. With traditional models of service, family and carers may transport a patient to a clinic appointment. With telehealth, they may instead be asked to fulfil some of the therapist’s role assisting with exercises or assessments at home. Local health professionals supporting telehealth consults may also necessitate taking on some of the roles usually performed by specialists. Conversely, new responsibilities may also be required of specialists, who may need to train, advise and assist local people on the ground in order to enlist their support. It is important for successful integration of the model that attention is paid to the existing skills of those who will be called on to undertake new tasks, and training provided where there is a lack of calibration between tasks and skill-sets.

Telehealth and rural allied health clinicians raised concerns about a number of *contextual integration* barriers to normalization of telehealth services. The latter in particular strongly felt that telehealth services do not currently fit well within existing health service structures, and identified numerous organizational and systemic barriers that would need to be overcome if telehealth is to be successfully integrated into existing mainstream health services. Telehealth clinicians also raised technological infrastructure as an issue that may inhibit contextual integration, including the challenges of dealing with rapidly changing technology and provision of ongoing technical support. Telehealth clinicians recognised that rapid and ongoing advances in technology will impact on relational integration and skill-set workability, posing challenges for the maintenance of the working knowledge required to effectively use telehealth. Lack of organizational support, jurisdictional constraints and incompatibilities across different organizational entities have also been recognised in the literature as barriers to implementation of telehealth [12, 13]. ‘Leadership support’ from health service decision makers and managers has been identified as a key factor in achieving broad implementation of telehealth, as such support is essential for addressing such contextual integration barriers raised by the health workers participating in this study [33].

This study indicates that both location of service (urban versus rural) and level of experience with telehealth do impact on health worker attitudes, and therefore the implementation potential of telehealth in the context of care for older people. As shown in Table 3, residential aged care staff working in rural facilities demonstrated, in NPT terms, high coherence, cognitive participation and collective action. In addition, their urban-based partners who provided the service, experienced telehealth practitioners, also showed that they engaged with and perceived the model to be a legitimate way of providing their service. These factors are likely to support embedding of this new mode of health delivery in the unique context of rehabilitation and geriatric outreach services to older people living in residential aged care.

Rural health practitioners’ attitudes regarding telehealth met many of the constructs outlined in NPT as necessary to successful implementation of new service models, despite these clinicians not having used the technology in the provision of their services. We argue that their awareness of the unmet healthcare needs of many in rural areas drives their engagement with the potential of telehealth and holds promise for integration of a rural telehealth service. However, our results also show that clinicians working in urban areas that are more easily able to see their patients face-to-face can also find telehealth models of service acceptable and willingly use the technology.

In contrast to urban clinicians with no experience of telehealth, the experienced telehealth clinicians in this study had significantly developed coherence, cognitive participation and collective action. They had re-conceptualised the way in which they provide their services through the lens of telehealth, rather than as a mere adjunct to conventional face-to-face therapy. They also had sufficient experience to develop insightful reflections on telehealth in rehabilitation, demonstrating *reflexive monitoring* in NPT terms. They were able to reflect on how they can further extend telehealth services and respond to challenges, by accessing and improving available technologies and pre-emptively managing risk. The telehealth clinicians were recruited to work on the telehealth trial from the cohort of urban clinicians, which supports existing evidence that experienced healthcare workers perceive telehealth services in a more positive light than those without telehealth experience [17, 19, 20].

However, it is possible that the clinicians who applied or were nominated to work on the telehealth trial had different characteristics from other staff. This study suggests that increasing exposure to the technology and experience providing telehealth may be beneficial but not sufficient to significantly enhance normalization potential. Novice providers of telehealth services displayed a burgeoning acceptance of telehealth, however they still conceptualised it within the bounds of conventional practice, rather than a distinct model of service. As put by Asch ‘The innovation that telemedicine promises is not just doing the same thing remotely that used to be done face-to-face but awakening us to the many things that we thought required face-to-face contact but actually do not’ [34]. We posit that a key difference between experienced telehealth and novice clinicians was this difference in conceptualisation, or in NPT terms, lack of coherence about telehealth on the part of novice clinicians. Hinging on this was a difference in expectation about how and the degree to which these clinicians will use telehealth. The former, by virtue of their involvement in a trial specifically evaluating telehealth, viewed telehealth as their core business. By contrast, novice clinicians perceived conventional face-to-face therapy as their core business, with telehealth incorporated at their discretion.

It remains to be seen whether these novice clinicians will further embrace and legitimise telehealth as their experience lengthens. This research suggests that in addition to exposure, attention needs to be paid to relational integration of the service. Researchers have highlighted the importance of understanding how telehealth dovetails with conventional services [14]. Analysis of the implementation of the Whole System Demonstrator trial in the UK, one of the largest trials of telehealth service provision, has demonstrated the challenges in achieving ‘whole system’ change and complete integration of telehealth services. Support of front-line healthcare staff is important to this process [35]. Wade et al. argue that clinician acceptance is the most crucial factor influencing successful and sustained implementation of telehealth services. Such acceptance can be enhanced by promotion of the efficacy, safety and normality of telehealth, focussing on relationship building within telehealth networks, disseminating evidence of the acceptability of telehealth, a comprehensive change management plan and adequate training and support [8, 13, 18, 36]. We posit that, as suggested by other researchers, accepting and enthusiastic clinicians will use the technology willingly [18]. However a comprehensive implementation strategy should also include development of a service framework, which explicitly defines the scope, position and use of telehealth within it, which should be clearly communicated to healthcare workers expected to use it.

### Limitations

As for qualitative research in general, the study is highly context specific. Further research in a range of rehabilitation, aged and palliative care contexts may highlight not only contexts in which clinician attitudes align with those found in our study, but also contexts in which clinicians attitudes and beliefs about telehealth may differ. Such research will help to further our understanding of the normalization of telehealth services in these health service areas.

The sampling approach and topic guide development used in this study were not originally designed for the purposes of analysis of implementation factors, or a NPT framework, but rather for the development of a related questionnaire on telehealth attitudes and preferences. Though the sample and topics covered in these focus groups were highly relevant to these areas, a more purposefully sampled study and theoretically derived interview guide may have produced more focussed results.

## Conclusions

Telehealth delivery approaches in the context of aged care services may be more readily normalized in areas where existing services are rationed or not accessible, such as rural areas. In this study, rural health clinicians and residential aged care staff were enthusiastic about the potential of telehealth to enhance healthcare access for their clients.

Experience and exposure to telehealth technology appeared to aid normalization, particularly among health workers providing services in less-rationed urban areas. However, changes in the way new interventions are conceptualised and perceived in relation to existing, conventional services may be more critical to attitudinal change and the normalization process. It will be important to show that telehealth is a feasible alternative to more traditional service delivery if we are to achieve widespread coherence and cognitive participation among staff expected to facilitate implementation and embedding of new telehealth services. The attention paid to necessary changes to organizational, systemic and technological infrastructure, as well as training and skill recalibration are also highlighted in this study as important factors to successful normalization of telehealth services in rehabilitation, aged and palliative care.

## Abbreviations

EP: Exercise physiology
NPT: Normalization process theory
OT: Occupational therapy
PT: Physiotherapy
RN: Rehabilitation nursing
SP: Speech pathology
SW: Social work
